# Genome-wide identification and functional prediction of long non-coding RNAs in Sprague-Dawley rats during heat stress

**DOI:** 10.1186/s12864-021-07421-8

**Published:** 2021-02-17

**Authors:** Jinhuan Dou, Flavio Schenkel, Lirong Hu, Adnan Khan, Muhammad Zahoor Khan, Ying Yu, Yajing Wang, Yachun Wang

**Affiliations:** 1 Key Laboratory of Animal Genetics, Breeding and Reproduction, MARA, National Engineering Laboratory for Animal Breeding, College of Animal Science and Technology, China Agricultural University, 100193 Beijing, People’s Republic of China; 2 Centre for Genetic Improvement of Livestock, Department of Animal Biosciences, University of Guelph, Guelph, Ontario N1G 2W1 Canada; 3 State Key Laboratory of Animal Nutrition, Beijing Engineering Technology Research Centre of Raw Milk Quality and Safety Control, College of Animal Science and Technology, China Agricultural University, 100193 Beijing, People’s Republic of China

**Keywords:** Heat stress response, LncRNAs, DEGs, Liver, Adrenal glands, Heat shock protein

## Abstract

**Background:**

Heat stress (HS) is a major stress event in the life of an animal, with detrimental upshots in production and health. Long-non-coding RNAs (lncRNAs) play an important role in many biological processes by transcriptional regulation. However, no research has been reported on the characterization and functionality of lncRNAs in heat-stressed rats.

**Results:**

We studied expression levels of lncRNAs in rats during HS, using strand-specific RNA sequencing. Six rats, three in each of Control (22 ± 1 °C) and H120 (42 °C for 120 min) experimental groups, were used to screen for lncRNAs in their liver and adrenal glands. Totally, 4498 and 7627 putative lncRNAs were identified in liver and adrenal glands of the Control and H120 groups, respectively. The majority of lncRNAs were relatively shorter and contained fewer exons than protein-coding transcripts. In total, 482 (174 up-regulated and 308 down-regulated) and 271 (126 up-regulated and 145 down-regulated) differentially-expressed lncRNAs (DElncRNAs, *P* < 0.05) were identified in the liver and adrenal glands of the Control and H120 groups, respectively. Furthermore, 1274, 121, and 73 target differentially-expressed genes (DEGs) in the liver were predicted to interact with DElncRNAs based on *trans*−/*cis*- and sequence similarity regulatory modes. Functional annotation analyses indicated that these DEGs were mostly significantly enriched in insulin signalling, myeloid leukaemia, and glucagon signalling pathways. Similarly, 437, 73 and 41 target DEGs in the adrenal glands were mostly significantly enriched in the cell cycle (*trans*-prediction) and lysosome pathways (*cis*-prediction). The DElncRNAs interacting with DEGs that encode heat shock proteins (HSPs) may play an important role in HS response, which include *Hsf4*, *Dnaja1*, *Dnajb4*, *Hsph1* and *Hspb1* in the liver, and *Dnajb13* and *Hspb8* in the adrenal glands. The strand-specific RNA sequencing findings were also further verified through RT-qPCR.

**Conclusions:**

This study is the first to provide a detailed characterization and functional analysis of expression levels of lncRNAs in liver and adrenal glands of heat-stressed rats, which provides basis for further studies on the biological functions of lncRNAs under heat stress in rats and other mammalian species.

**Supplementary Information:**

The online version contains supplementary material available at 10.1186/s12864-021-07421-8.

## Background

Heat stress (HS) is one of the main abiotic stressors that influence human and animal survival, welfare, and development [1–3]. Escalating global warming, combined with the global increase in the number of production animals and the intensification of agriculture [4, 5], has resulted in HS becoming a difficult challenge for livestock and poultry production. Heat stress leads to enormous economic losses to the livestock production industry [6], stemming from reduced production of meat, egg, and milk, decreased fertility, and increased morbidity and mortality [4, 7, 8]. The current trends in the increase of global temperature [9, 10] indicate that it is necessary and urgent to comprehensively investigate the genetic and biological mechanisms of HS, as well as develop long-lasting, cumulative, and significant strategies for preventing HS.

Over the past decades, HS research has been carried out in many species, such as humans [11], cattle [12], pigs [13, 14], corals [15], and rats [16, 17]. However, the regulatory mechanisms of HS are still unclear. Transcriptome sequencing technology for animals [18, 19] and cells [20] is becoming a suitable method for exploring HS-related genes and biological pathways. Studies have reported thousands of differentially-expressed genes (DEGs) under certain HS conditions [21–24]. There are many processes that affect the expression of genes, such as the regulation of long non-coding RNA (lncRNA) [25]. LncRNA is a non-coding RNA longer than 200 nucleotides in length and with more than two exons. LncRNA can regulate gene expression at the transcription and post-transcription levels [26]. Previous studies have reported several lncRNAs playing crucial role in HS response through interaction with transcription factors [27] or feedback regulation of key stress response proteins [28, 29]. Heat shock response is a major and crucial defence mechanism during HS, which contributes to cell recovery from heat shock damages, e.g., protein misfolding and aggregation [30, 31]. Furthermore, several lncRNAs have been identified in animals under HS conditions [32–34]. However, the understanding of the contributions of lncRNAs to the cellular HS response is still unclear.

The liver and adrenal glands play a key role in maintaining animal homeostasis during HS [19, 35, 36], but the role of lncRNA during this process still requires in-depth investigation. Therefore, the main aim of this study was to perform a transcriptomic analysis of rat liver and adrenal glands, following exposure to HS, to identify related DEGs, differentially-expressed lncRNAs (DElncRNAs), and key biological pathways related to HS response in rats. Our findings will contribute to a better understanding of the regulatory mechanisms of HS response in rats and other mammals.

## Results

### Comprehensive identification of lncRNAs in liver and adrenal glands

A total of ~ 29.9 and 28.3 million raw reads in the liver and adrenal glands were obtained (Additional file 2: Table S2), in which 29.8 and 28.1 million clean reads were aligned to the reference genome (Ensemble release version Rnor 6.0.91). The average mapping rate of clean reads in the liver and adrenal glands was 95.71 and 92.99%, respectively. Subsequently, 484,530 and 613,791 unique transcripts, both in liver and adrenal glands, were assembled from H120 and Control rats, respectively.

**Fig. 1:**
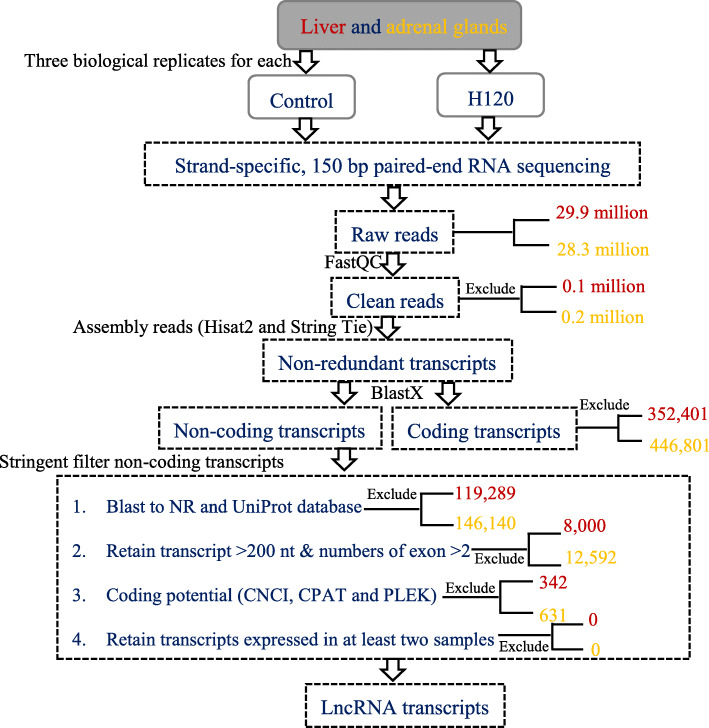
The detailed schematic pipeline of long-non-coding RNA (lncRNA) transcripts identification. Control was kept at room temperature (22 ± 1 °C, relative humidity [RH] (%): 50%); H120 were subjected to 42 °C and RH 50% for 120 min. NR: (National Center for Biotechnology Information) NCBI non-redundant (NR) protein database; UniProt: Universal Protein Resource; CNCI: coding-non-coding index; CPAT: the coding potential assessment tool; PLEK: predictor of lncRNAs and mRNAs based on the k-mer scheme

**Fig. 2:**
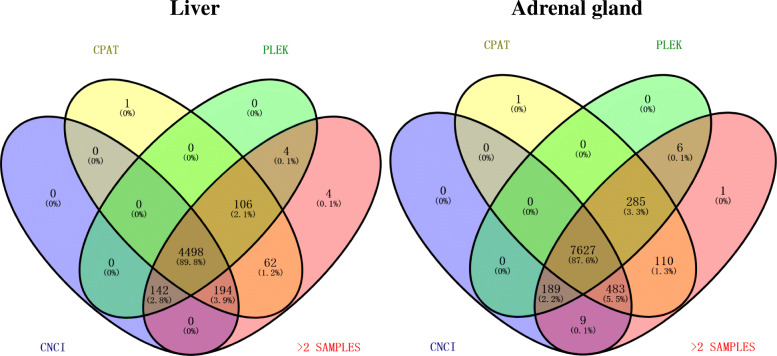
The Venn diagram for prediction of coding potential of non-coding transcripts in liver and adrenal glands. The > 2 SAMPLES means that only transcripts identified in at least two samples were retained for further analyses

Five filtering steps were performed for identifying candidate lncRNA (Fig. 1). Firstly, the assembled transcripts were filtered with rat coding gene sequences. Almost 72.72% (352,401) and 72.79% (446,801) of transcripts in liver and adrenal glands are coding genes, and the remaining 27.27% (132,129) and 27.21% (166,990) of transcripts are considered to be non-coding. Secondly, the transcripts that might encode conserved protein domains were further filtered out by comparing them to two protein databases including (National Center for Biotechnology Information) NCBI non-redundant (NR) protein database and Universal Protein Resource (UniProt) database and, as a result, 12,840 and 20,850 transcripts in the liver and adrenal glands were retained. LncRNAs are usually defined as non-coding RNAs longer than 200 nucleotides and having more than two exons. Based on these features, a third filter was applied, and 4840 (37.52%) transcripts in the liver and 8258 (39.61%) transcripts in the adrenal glands were removed. Finally, the coding-non-coding index (CNCI), the coding potential assessment tool (CPAT), and the predictor of lncRNAs and mRNAs based on the k-mer scheme (PLEK) were used to evaluate the protein-coding potential, and 4498 and 7627 transcripts in the liver and adrenal gland tissues were retained (Fig. 2). After employing the four above mentioned stringent filters, transcripts expressed only in one sample were also removed. Finally, 4498 and 7627 transcripts in the liver and adrenal gland tissues were considered as putative lncRNAs (Fig. 2).

### Classification and characterization of lncRNAs in liver and adrenal glands

**Fig. 3:**
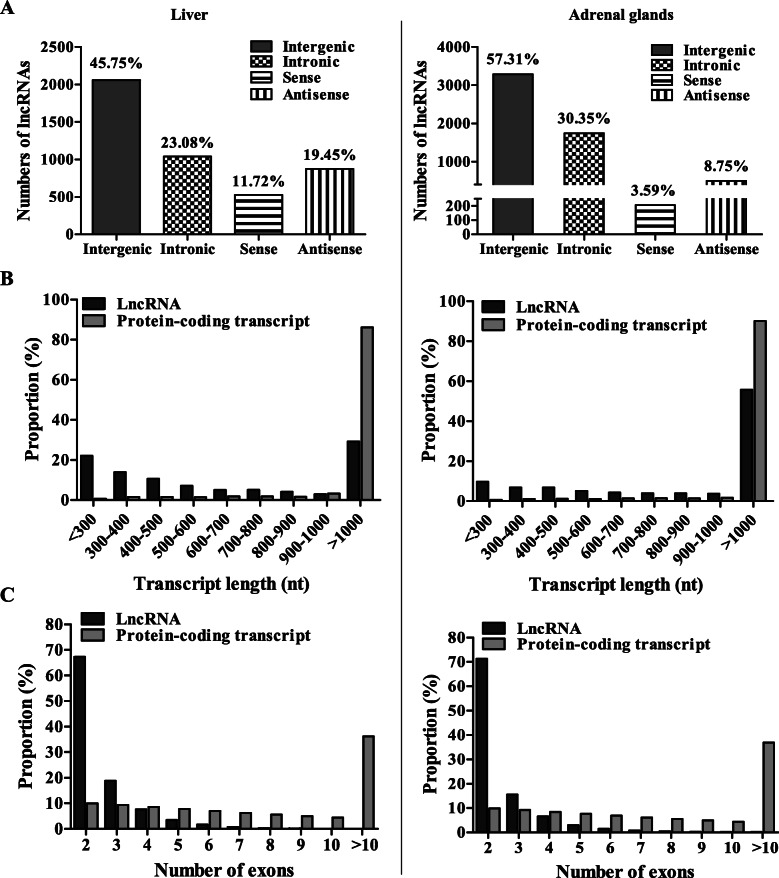
The classification and characterization of lncRNAs identified in liver and adrenal glands. **a** Number of lncRNAs in different categories. **b** Transcript lengths of protein-coding transcripts and lncRNAs. **c** Number of exons per transcript for protein-coding transcripts and lncRNAs. Left panel depicts results for liver and right panel for adrenal glands

According to the location relative to the nearest protein-coding gene (PCG), lncRNAs in the liver and adrenal glands were further classified into four types, including intergenic, intronic, sense, and antisense (Fig. 3a). About 45.75% of lncRNAs in the liver (Fig. 3a_left panel) and 57.31% of lncRNAs in the adrenal glands (Fig. 3a_right panel) were located in intergenic regions, whereas 23.08 and 30.35% lncRNAs were transcripts most from introns. In addition, 19.45% of lncRNAs in the liver were antisense of PCGs, which were more frequent than those lncRNAs that overlapped with genes (11.72%). The same feature was also found in adrenal glands, i.e. the number of antisense lncRNAs was 2.44 times greater than that of sense lncRNAs (Fig. 3a_right panel).

Figure 3b and c show the transcript length and number of exons of lncRNAs compared to protein-coding transcripts. Figure 3b shows that almost 70.8% of lncRNAs in the liver ranged in size from 200 to 1000 nucleotides, with only 29.20% > 1000 nucleotides. In contrast, about 86.15% of protein-coding transcripts were > 1000 nucleotides (Fig. 3b_left panel). In the adrenal glands, similar characteristics of lncRNAs and protein-coding transcripts were observed with 55.79% of lncRNAs having > 1000 nucleotides and 90.09% of protein-coding transcripts having> 1000 nucleotides (Fig. 3b_right panel). Interestingly, most of the lncRNAs of the liver and adrenal glands (86.11 and 86.89%, respectively) contained two to three exons, while the number of exons of protein-coding transcripts ranged from two to over ten (Fig. 3c). These statistics indicated that the majority of lncRNAs were relatively shorter and contained fewer exons than protein-coding transcripts.

### Identification of temperature-dependent differentially-expressed lncRNAs (DElncRNAs)

**Fig. 4:**
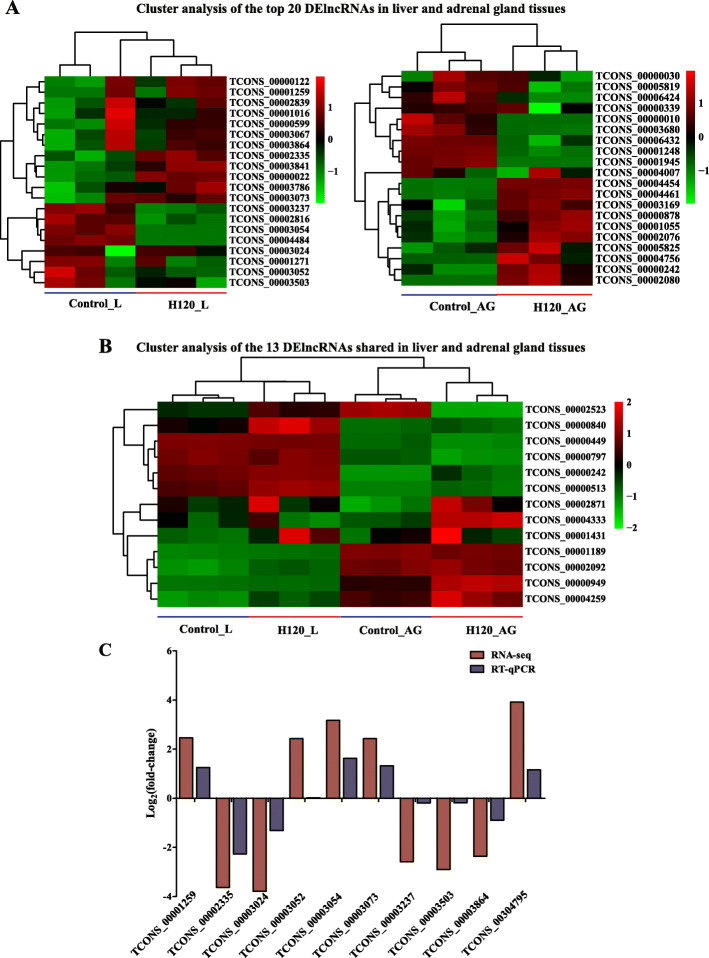
Hierarchical clustering and validation analysis of the specific differentially-expressed lncRNAs (DElncRNAs). **a** The Pheatmap of the top20 DElncRNAs in liver and adrenal glands. **b** The Pheatmap of commonly identified DElncRNAs in liver and adrenal glands. **c** The comparative analysis of the expression level of randomly selected lncRNAs in liver and adrenal glands using RNA-seq and RT-qPCR. The log _(10 + 1)_-transformed FPKM values of DElncRNAs (rows) are clustered using hierarchical clustering, and the samples are grouped according to the similarity of expression profiles of DElncRNAs

A total of 482 and 271 DElncRNAs (*P* < 0.05) in the liver and adrenal glands were obtained and further divided into six categories according to fold change (FC) values (Table 1 and Additional file 3: Table S3). The top 20 DElncRNAs in the liver (12 up-regulated and 8 down-regulated) and adrenal glands (11 up-regulated and 9 down-regulated) were used for clustering analyses, which indicated clearly-clustered results (Fig. 4a). Three samples (rats) for each treatment group were clustered together. Additionally, 13 DElncRNAs were shared between the liver and adrenal glands (Fig. 4b), 469 and 258 DElncRNAs were identified in the liver and adrenal glands, respectively, as having tissue-specific expression. Among which, most lncRNAs (63.54%) were down-regulated in the liver, and over half (54.6%) of lncRNAs were down regulated in the adrenal glands. The log-transformed relative expression FC of ten lncRNAs in H120 and Control groups generated from real-time quantitative PCR (RT-qPCR) were in line with the results of RNA-seq data (Fig. 4c). The Pearson correlation coefficient (PCC) between RT-qPCR and RNA-seq was as high as 0.88, which confirmed the reliability of the RNA-seq analysis.

**Table 1:** Statistical summary of number of lncRNAs (DElncRNAs) identified in liver and adrenal gland tissues in H120 vs. Control groups

Criteria	Expression models	Liver	Adrenal glands
		DElncRNAs (*P* < 0.05)	DElncRNAs (*P* < 0.05)
No filtering of FC	Total	482	271
	Up	174	126
	Down	308	145
|FC| > 2	Total	122	173
	Up	61	85
	Down	61	88
|FC| > 4	Total	34	78
	Up	20	42
	Down	14	36
|FC| > 5	Total	17	57
	Up	10	32
	Down	7	25
|FC| > 8	Total	7	42
	Up	2	20
	Down	5	22
|FC| > 10	Total	5	34
	Up	1	15
	Down	4	19

Total means the total number of differentially expressed lncRNAs (DElncRNAs, *P* < 0.05). Up means the up-regulated DElncRNAs in liver and adrenal glands when comparing H120 vs. Control groups. Down means the down-regulated DElncRNAs in liver and adrenal glands when comparing H120 vs. Control groups

*FC* fold change

### Functional prediction of DElncRNAs

#### Construction of co-expression network between DElncRNAs and target DEGs

**Fig. 5:**
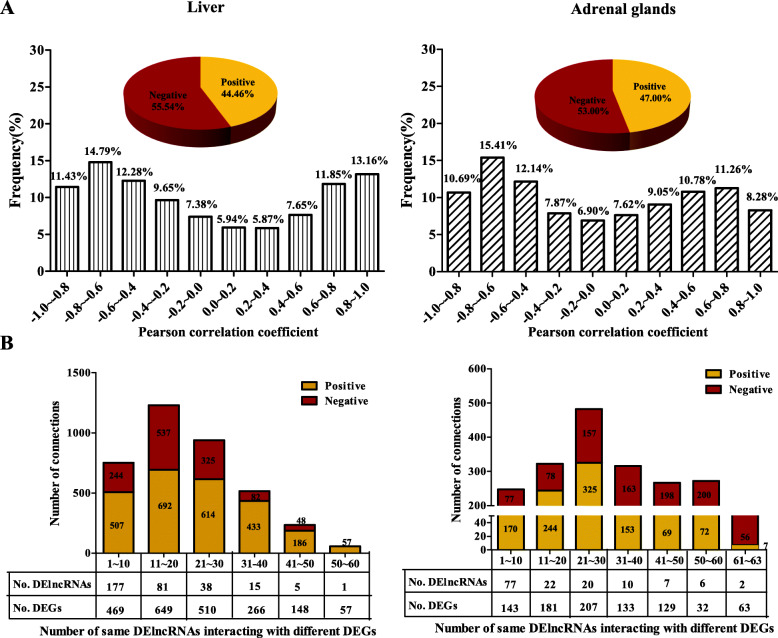
Statistical analysis of co-expression and the classification of the correlation frequency between DElncRNAs and differentially-expressed genes (DEGs). **a** Pearson correlation coefficient (PCC) analyses between DElncRNAs and DEGs in liver and adrenal glands. **b** Statistical analysis of the number of DEGs related to the same DElncRNAs

A total of 3909 and 4953 DEGs (*q* < 0.05) were identified in rat liver and adrenal glands in a previous study [22]. The co-expression network between DElncRNAs and DEGs in the liver and adrenal gland tissues was created (Fig. 5). 1,935,712 connections between DElncRNAs and DEGs in the liver were identified, in which 44.46% were positive connections, and 55.54% were negative connections (Fig. 5a_left panel). Furthermore, among all relationships, the PCCs of 14.79% were between − 0.8 and − 0.6 and followed by 13.16% connections between 0.8 and 1.0. In the adrenal glands, 1,492,397 links were identified between DElncRNAs and DEGs; the positive and negative associations were 47.00 and 53.00%, respectively. Moreover, most PCCs between DElncRNAs and DEGs in the adrenal glands (15.41%) ranged from − 0.8 to − 0.6, followed by 12.14% PCCs between − 0.6 and − 0.4 (Fig. 5a_right panel). In order to better indicate the relationship between the DElncRNAs and DEGs, the connections with high correlation |PCC| > 0.99 were selected for further analyses (Fig. 5b). Three thousand seven hundred twenty-five connections including 317 DElncRNAs and 1274 DEGs, and 1969 connections including 139 DElncRNAs and 437 DEGs in the liver and adrenal glands were retained (Additional file 4: Table S4). All connections between DElncRNAs and DEGs were then divided into 6 or 7 categories in the liver and adrenal glands, respectively (Fig. 5b). The largest number of connections between DElncRNAs and DEGs in the liver was identified in the cluster of one DElncRNAs interacting with 11 ~ 20 DEGs, which includes 81 unique DElncRNAs and 648 DEGs. Only one DElncRNA (TCONS_00000716) was found to interact with 57 DEGs when HS occurred. Four hundred eighty-two connections in the adrenal glands were clustered in the classification of one DElncRNA interacting with 21 ~ 30 DEGs, which includes 20 unique DElncRNAs and 207 unique DEGs. Therefore, Fig. 5 shows that multiple lncRNAs might regulate one DEG and, on the contrary, multiple DEGs may be regulated by a single lncRNA.

The functions of 1274 and 437 DEGs interacting with 317 and 139 DElncRNAs in the liver and adrenal glands were annotated (Additional file 5: Table S5 and Additional file 6: Figure S1A). In the liver, 1274 DEGs were significantly enriched (*P* < 0.05) in 124 biological process (BP) terms, such as response to heat (GO: 0009408), response to hypoxia (GO: 0001666), response to unfolded protein (GO: 0006986), and biosynthesis and metabolism of glucose and fat acid (e.g., GO: 0042593, GO: 0006633 and GO: 0071397). The Kyoto Encyclopedia of Genes and Genomes (KEGG) analysis showed 20 significantly enriched pathways (*P* < 0.05) in liver (Additional file 6: Figure S1A_left panel), some of which were associated with glucose and fat acid metabolism (e.g., adipocytokine signaling pathway), hormone regulation (e.g., estrogen signaling pathway), and cancer pathways (e.g., PPAR signaling pathway), suggesting that HS response may be a complex process comprising of neurohormonal regulation, energy metabolism, and immune response. Twenty-six BPs were significantly enriched (*P* < 0.05) by 437 DEGs in the adrenal glands (Additional file 5: Table S5), with three of them shared with liver, i.e. glycosaminoglycan biosynthetic process (GO: 0006024), protein phosphorylation (GO: 0006468) and cellular response to amino acid starvation (GO: 0034198). Furthermore, five significant pathways (*P* < 0.05) were detected (Additional file 6: Figure S1A_right panel), but none of them were shared in the liver.

#### *Cis*-prediction of DElncRNAs

A total of 512 and 545 genes were predicted in the liver and adrenal glands, with 121 and 191 DEGs (Additional file 7: Table S6). Functional annotation of all the predicted genes (Additional file 8: Table S7) and only the DEGs (Additional file 9: Table S8 and Additional file 6: Figure S1B**)** were performed. In the liver, 121 DEGs were significantly enriched (*P* < 0.05) in 13 BPs, including the radial glial cell differentiation (GO: 0060019) with the highest fold enrichment score of 67.44, followed by CDP-choline pathway (GO: 0006657) and JAK-STAT cascade involved in growth hormone signaling pathway (GO: 0060397). All DEGs in the liver were enriched in five pathways, and only one pathway, acute myeloid leukemia (rno05221), was significantly enriched (*P* < 0.05) under the H120 treatment. In the adrenal glands, 33 BPs (Additional file 9: Table S8), as well as four pathways [e.g., lysosome (rno04142), peroxisome (rno04146), gap junction (rno04540) and NF-kappa B signaling pathway (rno04064)], were significantly enriched (*P* < 0.05). Furthermore, the NF-kappa B signaling pathway has been shown to play a crucial and major role during heat stress response through activating autophagy [37].

#### Identification of DElncRNAs & DEGs interaction based on similarity search method

In order to perform the functional prediction for the DElncRNAs more comprehensively, the potential lncRNA-mRNA interactions based on the similarity-search method was investigated. Overall, 17,251 potential RNA-mRNA interactions in the liver were detected between 1180 DElncRNAs and 364 genes, and 9917 potential RNA-mRNA interactions between 1985 DElncRNAs and 171 genes were identified in the adrenal glands (Additional file 10: Table S9). In the liver, functional enrichment analysis of the 364 genes revealed 28 significantly enriched BPs (*P* < 0.05), which were mainly engaged in cell proliferation, positive regulation of GTPase activity and vesicle-mediated transport (Additional file 11: Table S10). For the KEGG analysis, eight pathways were detected, two of which are related to cellular growth and development (*P* < 0.05; Additional file 6: Figure S1C_left panel). Furthermore, 26 BPs were identified in the adrenal glands (*P* < 0.05), with some pathways positively regulating the transcription from the RNA polymerase II promoter, positively regulating transcription, DNA-template, and stimulating cell and neuron development (Additional file 11: Table S10). Twenty significantly regulated pathways were detected in the adrenal glands (Additional file 6: Figure S1C_right panel), 15 of them are directly engaged in different types of cancer, suggesting that innate immunity and inflammation-related pathways in the adrenal glands could be mobilized to respond to HS. In addition, pathways such as the MAPK signaling pathway [38] and the GnRH signaling pathway [39] have been reported in previous HS studies.

By combining the DEGs identified in the current study, 73 (48 up-regulated and 25 down-regulated) and 41 (12 up-regulated and 29 down-regulated) target genes in the liver and adrenal glands were differentially expressed. Functional annotation of these DEGs is shown in Additional file 12: Table S11. Seventy-three DEGs in the liver were significantly enriched (*P* < 0.05) in the process of muscle development, such as striated muscle contraction (GO: 0006941), myosin filament assembly (GO: 0031034), muscle contraction (GO: 0006936), and forebrain development (GO: 0030900). Whilst, these DEGs were also significantly involved (*P* < 0.05) in the pathways of glucagon signaling and tight junction. Functional annotation for the 41 DEGs obtained in the adrenal glands revealed that the positive regulation of axon regeneration (GO: 0048680), which showed the highest fold enrichment score of 106.27, was significantly enriched (*P* < 0.05) and the DEGs *Ndel1* and *Braf* were both up-regulated. Furthermore, the other significantly-enriched BP term was phospholipid translocation (GO: 0045332) and the enriched genes *ATP10a* and *ATP8b5p* were all down-regulated. A remarkable finding was the significantly-enriched (*P* = 0.026) cellular component (CC) term of extracellular exosome (GO: 0070062) with 11 genes in the adrenal glands being part of the extracellular exosome, of which four genes were up-regulated and seven genes were down-regulated (Additional file 12: Table S11). No pathway was significantly enriched in the adrenal glands.

### The interaction between DElncRNAs and DEGs encoding the heat shock proteins (HSPs)

**Fig. 6:**
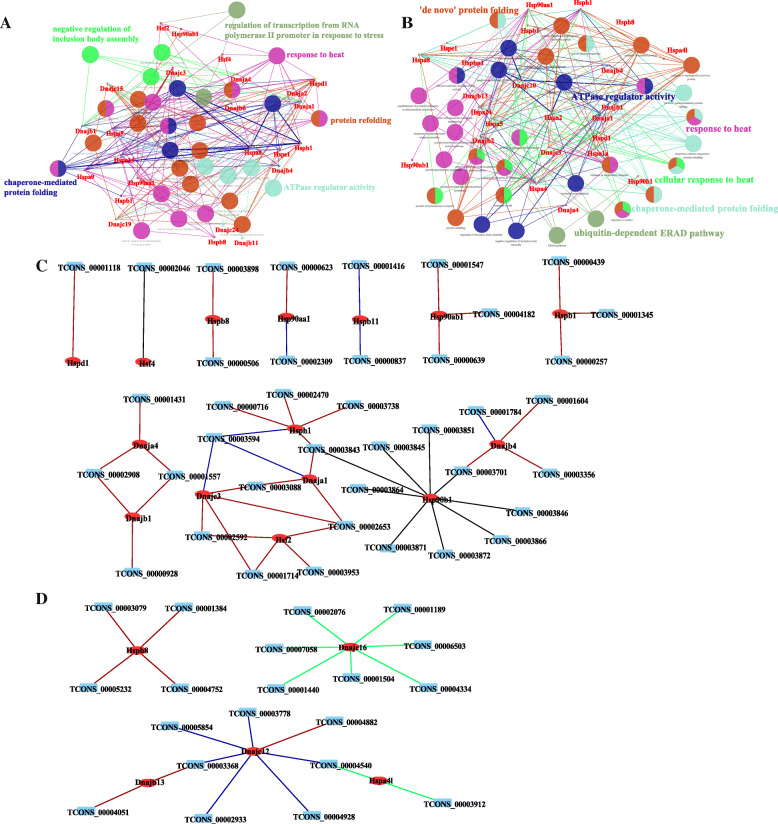
Functional annotation of DEGs encoding proteins of the heat shock protein (HSP) family and their potential interactions with DElncRNAs. **a** and **b** Functional interaction networks among various DEGs that encode heat shock proteins (HSPs) identified in liver (**a**) and adrenal gland (**b**) tissues. **c** and **d** Interaction networks among different HSP-encoding DEGs and DElncRNAs. Interactions between DEGs and DElncRNAs were predicted by *trans*-, *cis*- and sequencing similarity regulatory module. The interaction network was visualized by Cytoscape software. For the nodes, the red ovals mean DElncRNAs, blue rectangles mean DEGs; for the lines, the dark orange lines mean positive correlation between DEGs and DElncRNAs (*trans* prediction), the blue lines mean negative correlation between DEGs and DElncRNAs (*trans* prediction), the black lines mean DElncRNAs regulated DEGs by *cis*-action and the green lines mean DElncRNAs regulated DEGs based on the sequence similarity

Heat stress may induce misfolding and aggregation of proteins, halt the whole proteins translation, and further cause the apoptosis of cells [31]. The HSP/chaperone network is a major component of multiple stress responses, which is recruited under HS and manage protein folding [30]. From a previous study, 30 and 33 genes of the HSP family genes in the liver and adrenal glands were differentially expressed, with fold change ranging from 0.19 to 28.03 and from 0.11 to 183.84 [22]. All of these 30 HSP encoding genes in the liver were significantly enriched (*P* < 0.05) in 5 BP terms, such as chaperone-mediated protein folding (GO: 0061077), response to heat (GO: 0009408), ATPase regulator activity (GO: 0060590), protein refolding (GO: 0042026), and regulation of transcription from RNA polymerase II promoter in response to stress (GO: 0043618) (Fig. 6a). The 33 genes in the adrenal glands were significantly enriched (*P* < 0.05) in 6 BP terms, and 2 of them (GO: 0061077 and GO: 0009408) were shared with liver tissue (Fig. 6b). The predictive analysis of target genes for the lncRNAs also displayed 19 and 6 HSP encoding genes in the liver and adrenal glands, respectively (Fig. 6a). Among which, 15 and 5 DEGs in the liver and adrenal gland tissues were regulated by DElncRNAs (Fig. 6c and d). The interactions among different HSP encoding DEGs and DElncRNAs indicate that lncRNAs play a crucial role in post-transcriptional regulation of HS-related genes.

## Discussion

Over the past decades, with the discovery of few new classes of regulatory non-coding RNAs, an increasing amount of evidences reveals that lncRNAs are engaged in many BPs, such as transcriptional [40, 41] and epigenetic regulation [42, 43], regulation of cell growth [44, 45] and those contributing towards disease etiology [46]. The HS response process is no exception, as lncRNAs are being identified as mediators of general transcription repression of genes [47, 48]. However, a comprehensive analysis of the profiles of lncRNAs showing differential expression under low and mild HS conditions has not yet been reported. In the present study, a well-established HS-rat model was used to explore the expression profile and potential functions of lncRNAs involved in the HS response process in rat liver and adrenal glands by strand-specific RNA-seq. Notably, this study investigated for the first time the expression pattern of lncRNAs in adrenal glands under HS treatment. A total of 4498 and 7627 transcripts were identified in the liver and adrenal glands, which were considered as putative lncRNAs (Figs. 1 and 2). The majority of lncRNAs in the liver and adrenal glands is located in intergenic regions, which is consistent with previous studies performed in other mammalian [49, 50], plant [51, 52] and fungal [53, 54] species. In addition, previous research showed that animal lncRNAs are shorter and have fewer exons than PCGs [55, 56], which is in line with our findings (Fig. 3b and c).

LncRNAs have been reported to be involved in regulation of the HS response in some species [19, 34, 57, 58]. In our analysis, 482 (174 up-regulated and 308 down-regulated) and 271 (126 up-regulated and 145 down-regulated) DElncRNAs in rat liver and adrenal glands were identified under H120 condition, respectively (Table 1). Out of them, 13 DElncRNAs were shared in the liver and adrenal gland tissues (Fig. 4b), which might be meaningful for exploring the regulatory mechanism between the hypothalamic-pituitary-adrenal axis (HPA axis) and liver under HS. It has been reported that lncRNA modulate the transcription of genes by several different actions, including *trans*-regulatory, in which lncRNAs themselves co-regulate their distant genes; *cis*-regulatory, in which lncRNAs control the expression of their neighbouring genes; or they interact with the pre-mRNA based on the sequence similarity [26, 50, 59]. Therefore, in order to further demonstrate the potential function of lncRNAs in the HS process, the putative *tran-* and *cis-*regulatory, as well as sequencing similarity regulatory module, were analysed. Furthermore, all the 3909 and 4953 DEGs in the liver and adrenal glands were used as targets for DElncRNAs and performed functional annotation for the DEGs that met various rules of function prediction (Additional file 6: Figure S1 and Additional file 5: Table S5, Additional file 8: Table S7, Additional file 9: Table S8, Additional file 11: Table S10, Additional file 12: Table S11). The findings are consistent with results of our previous study [60] and other literature reports [54]. Furthermore, the shared pathways produced from target DEGs in the liver and adrenal glands might contribute to the understanding the potential crosstalk mechanisms between two tissues under HS treatment [61].

The importance of HSP family in regulating the process of the HS response has been highlighted in very early studies [62, 63]. The protection provided by HSPs gives ability for organisms to cope with HS at high temperatures. Furthermore, previous studies have discovered the crucial role of several lncRNAs in the regulation of the HS response [64, 65]. For instance, the drosophila hsr-omega (hsrω) non-coding RNA, which might be the most extensively studied lncRNA, is induced by heat shock [66–68]. The heat shock RNA-1 (HSR1) is one of the earliest reported lncRNAs participating in the HS response by regulating the activation of HSF1 [69]. In stress-free cells, HSF1 can bind to some chaperones, such as HSPC1/HSP90. When HS occurs, HSF1 is released and binds to heat shock elements in the HSP encoding gene promoter, thereby inducing its transactivation [64]. In summary, it is important to investigate the impact of lncRNAs on HS response from the perspective of heat shock response. In the present study, the DEGs that encode HSPs were functionally annotated (Fig. 6a) and participated in interaction networks with DElncRNAs by *trans*-, *cis*- and similarity regulatory analyses (Fig. 6b). Two HSF genes, *Hsf4* and *Hsf2*, in the liver were found to interact with one (TCONS_00002046) and four (TCONS_00002592, TCONS_00001714, TCONS_00003953 and TCONS_00003953) DElncRNAs by *cis*- and *trans*-action, respectively. Furthermore, the *Hsf4* was down-regulated (*q* < 0.05) with a FC greater than five after H120. It has been reported that *Hsf4* can interact with the canonical heat shock element of the alphaB-crystallin gene (*Hspb6* in rat), whilst, *Hsf2* is heat-inducible and functions in heat shock-induced autophagic cell death [70, 71]. In addition, among all the DEGs encoding HSPs with a fold change greater than 5, the *Dnaja1*, *Dnajb4, Hsph1*, *Hspb1* genes in the liver, and *Dnajb13* and *Hspb8* genes in the adrenal glands are all known to play diverse roles in HS regulation [21, 72–75]. The DElncRNAs that interacted with DEGs encoding HSPs are worth exploring further to find out the specific role of lncRNAs in mRNA transcription and post-transcriptional regulation during HS.

## Conclusions

Many lncRNAs involved in the HS response in rat liver and adrenal gland tissues were discovered and characterized, and their potential *cis*-, *trans*-, and sequencing similarity acting functions were predicted based on their corresponding target DEGs. These findings provide a catalogue of rat liver and adrenal glands lncRNAs and will contribute to a better understanding of the regulatory mechanisms of HS responses in rats and other mammalian species. Future studies are required to explore the physiological functions and the mechanisms by which these lncRNAs respond to HS.

## Methods

### Animals and treatments

In a previous research [22], a total of 99 eight-week-old pathogen-free female Sprague-Dawley rats (Beijing Vital River Laboratory Animal Technology Co., Ltd., Beijing, China), weighing 205 ± 7.16 g, were used to establish HS model under various HS conditions. Eighteen of them were assigned to the HS treatment (H120; 42 °C for 120 min). Ten rats, including 5 rats from Control group and 5 from the H120 group were used to perform the RNA-seq. Based on the PCC of samples, 3 rats in each group were selected for lncRNA-seq. Prior to the beginning of the HS experiment, all experimental rats were housed (3 rats per cage) at 22 ± 1 °C and 50% room humidity with the light/dark cycle (on 06:00 AM, off 18:00 PM) for 1 week. During the whole experiment, rats were given access to feed and water ad libitum. Moreover, the experiment was performed in the floor-standing artificial climate incubator (BIO250, BOXUN Medicine Instrument Co, Shanghai, China). Rats in the Control group were never introduced to the incubator and were placed at room temperature. The experiment was conducted with healthy and conscious rats.

### Tissue collection and RNA extraction

The experimental rats were anesthetized by intraperitoneal injection of 1%, 1.2 mL sodium pentobarbital (40 mg/kg of body weight) [76]. After 2 min, all anesthetized animals were immediately euthanized by cardiac blood collection just after loss of consciousness. The protocols of blood samples collection were reported previously [77, 78]. Briefly, the hair on the abdomen of animals was cut with sterile surgical scissors (Shinva Medical Instrument Co.Ltd., Shandong, China) and the skin was disinfected with 75% alcohol. Then, rats were held with left hand and kept in dorsal recumbency position at an approximately 30° angle (head lowermost). A #4 needle was inserted into the rat’s heart by the right hand at the 3rd and 4th intercostal space. About 4 mL blood sample was collected, the rat’s abdomen was quickly dissected by sterile surgical scissors and forceps (Shinva Medical Instrument Co.Ltd., Shandong, China). Then the liver and adrenal glands were collected, washed in ice-cold phosphate buffer solution (PBS) and snap-frozen immediately in liquid nitrogen. Total RNA of the liver and adrenal glands was extracted using RNA regent (HUAYUEYANG Biotechnology (Beijing) Co. Ltd) according to the manufacture’s protocol. NanoDrop 2000 (Thermo Fisher Scientific, Waltham, MA) was used to measure the concentrations and purity of RNA. The RNA Nano 6000 Assay Kit of the Agilent Bioanalyzer 2100 system (Agilent Technologies, CA, USA) was employed to assess the integrity of RNA integrity.

### Transcriptome library construction and paired-end strand-specific transcriptome (rRNA-free) sequencing

The protocols of transcriptome library construction and paired-end strand-specific transcriptome sequencing were previously reported [79]. Briefly, 3 μg RNA per sample was used for RNA-seq library construction. Firstly, due to some lncRNAs lacking the poly (A) tail, Epicentre Ribo-zero™ rRNA Removal Kit (Epicentre, USA) was used to remove the ribosomal RNA from the total RNA, and ethanol precipitation was used to clean up the rRNA free residue. Subsequently, the rRNA-depleted RNA by NEBNext® Ultra™ Directional RNA Library Prep Kit for Illumina® (NEB, USA) was used to produce sequencing libraries according to the manufacturer’s recommendations. Then, the random hexamer primer and M-MuLV Reverse Transcriptase (RNaseH-) were employed to synthesize the first-strand cDNA, and the DNA Polymerase I and RNase H was subsequently used to synthesize the second-strand cDNA. The 3′ ends of DNA fragments was adenylated and NEBNext Adaptor with hairpin loop structure was ligated to prepare for hybridization. With the purpose of selecting cDNA fragments of favorably 250 ~ 300 bp in length, the AMPure XP system (Beckman Coulter, Beverly, USA) was selected to purify the library fragments. Before PCR experiment, 3 μL USER Enzyme (NEB, USA) was used with size-selected, adaptor-ligated cDNA at 37 °C for 15 min followed by 5 min at 95 °C. In the PCR experiment, the Universal PCR primers, Index (X) Primer and Phusion High-Fidelity DNA polymerase were added in the reaction system. At last, AMPure XP system and the Agilent Bioanalyzer 2100 system was used to purify the products and evaluate the library quality, respectively. A cBot Cluster Generation System using HiSeq 4000 PE Cluster Kit (Illumina, NEB, USA) was used to cluster the index-coded samples following the manufacturer’s instructions. Subsequently, an Illumina Hiseq 4000 platform was used to sequence the library preparations and produced 150 bp paired-end reads.

### Transcriptome library construction and paired-end RNA-sequencing

In a previous research [22], the mRNA profiles in the liver (*n* = 5) and adrenal glands (*n* = 5), including the same samples of liver and adrenal glands sequenced by strand-specific transcriptome sequencing in the current study, were analyzed using the RNA-seq technique. Briefly, the library was sequenced in a paired-end reads modus of 150 bp per read using Illumina® HiSeq 2000 platform. Furthermore, in this previous study, genome-wise false discovery rate (FDR), termed as q-value was calculated for differential expression analysis, and genes with *q* < 0.05 were selected as DEGs [22].

### Quality control for sequencing data and assembly of RNA transcripts

All sequencing reads were quality checked and trimmed to remove reads containing adapters, reads with more than 10 Ns and low-quality reads (i.e., more than 50% of the reads with a quality score of less than 10 or read length < 30) from raw data. The quality of clean data was assessed via the software of FastQC version 0.11.7 [80]. At the same time, Q20, Q30, and GC content of the clean data were calculated. All the downstream analyses were based on clean data with high quality. The clean reads were aligned with the rat reference genome (Ensemble release version Rnor 6.0.91, ftp://ftp.ensembl.org/pub/release-95/genbank/rattus_norvegicus/) using Hisat2 version 2.1.0 [81]. Reads met with the minimum isoform fraction 0.01 and minimum reads per bp coverage were assembled and quantified using String Tie version 1.3.6 [82]. The fragments per kilobase of exon model per million reads mapped (FPKM [controlling for fragment length and sequencing depth]) values were used to estimate the expression of genes and transcripts.

### Bioinformatics analysis for identification of LncRNA

All transcripts were divided into PCGs and non-coding transcripts after the reference genome annotation. A non-coding transcript that overlapped with PCGs, shorter than 200 nucleotides and containing single exon were filtered out. Then filtered transcripts were aligned against to the NCBI NR database (www.ncbi.nlm.nih.gov/protein) and UniProt rat_10116 protein database (www.uniprot.org) [83]; any transcripts which shown sequence similarity with any of these proteins with a cut-off E value of 10^− 5^ were removed. After these two filtering steps, the software CNCI version 2 [84], PLEK version 1.2 [85], CPAT version 2.0 [86] were used to predict the coding potential of transcripts. The candidate transcripts with no putative coding potential (CNCI score < 0), PLEK score < 0, and coding probability cut-off value of CPAT < 0.44 were considered as final lncRNAs. Furthermore, the final lncRNAs only identified in at least two samples were used for further analyses.

### Classification and characteristic analysis of LncRNAs

The identified lncRNAs were further classified as intergenic, intronic, sense, and antisense lncRNAs based on the spatial relationships of their transcripts loci with PCGs using Cuffcompare tool [50]. The transcripts length and exon number for protein-coding transcripts and lncRNA transcripts were assessed [52]. The proportion of different kinds of lncRNAs and protein-coding transcripts was calculated.

### DElncRNAs between H120 and control

The identification of DElncRNAs in the liver and adrenal glands between H120 and Control was carried out using Ballgown package of R version 3.5.3 (TUNA Team, Tsinghua University, Beijing, China). The FC of lncRNAs were calculated as log_2_ (FPKM H120/FPKM Control) and lncRNAs with *P* < 0.05 were identified as DElncRNAs.

### Validation of LncRNAs transcripts by real-time quantitative PCR (RT-qPCR)

A total of 2 mL RNA of the liver were transcribed into cDNA using the cDNA Synthesis SuperMix kit (Trans, Beijing, China). Ten lncRNAs, including nine top 20 DElncRNAs and one non-significantly expressed lncRNA, but with high expression levels, were randomly selected to carry out the RT-qPCR. The glyceraldehyde-3-phosphate dehydrogenase (*GAPDH*) was used as the internal standard to normalize the expression level of target genes. Primers for *GAPDH* and lncRNAs were designed using Primer-BLAST (www.ncbi.nlm.nih.gov/tools/primer-blast/index.cgi?LINK_LOC=BlastHome) [87] and double-checked by Oligo v7 (Molecular Biology Insights, Inc., Cascade, CO, USA). All the primers were synthesized by Shanghai Sangon Biotech Co., Ltd. (Shanghai, China) and were specified in Additional file 1: Table S1. Each reaction was performed in 20 mL mixtures, including 2 mL diluted cDNA sample as template, 10 mL SYBR Premix Ex Taq (2х) (TaKaRa, Kyoto, Japan), 1 mL forward and 1 mL reverse gene-specific primers and 6 mL ddH_2_O. The PCR reaction procedure comprised an initial degeneration at 95 °C for 10 min, 45 cycles of degeneration at 95 °C for 10 s, annealing at 58 °C for 15 s, and extension at 72 °C for 15 s, followed by a final extension at 72 °C for 30 s. The comparative threshold cycle (Ct) value method was adopted to analyze the relative gene expression. Triplicate RT-qPCRs were accomplished on each cDNA. RNA expression levels relative to the *GAPDH* gene were calculated as 2^-△△Ct^ according to previous research [88]. In order to compare the results of the RNA-seq analysis and RT-qPCR, fold change values were log_2_ transformed.

### Investigation of *trans*/*cis*/binding nuclei-target genes’ regulation and functional annotation of the target genes

Three methods were used to predict the target genes of the DElncRNAs based on the different action modes of lncRNA. One was *trans-*prediction [89], in which the principle of prediction was the co-expression relationship between lncRNA and their target protein-coding genes. Pearson correlation coefficient between expression patterns were calculated by R software, using the expression values of DElncRNAs, target genes, and target DEGs identified in our previous research [22]. In order to show a more accurate and intuitive relationship between DElncRNAs and DEGs, only highly correlated (|PCC| > 0.99) expressions were selected to construct the interaction network. The other action mode analyzed was *cis*-prediction [90], which was based on the adjacent positional relationship between DElncRNAs and target genes. The DElncRNA-mRNA pairs located on the same chromosome within 100 kb were identified as potentially *cis*-regulated. This analysis was completed using the BioMart tool of Ensemble genome browser 98 online (http://useast.ensembl.org/biomart/martview/8cae5041d3bb22709301ea05f556fc84) [91]. The last prediction was based on the similarity-search method. The detailed analysis process was according to a previously published paper [50]. All the predicted target genes of DElncRNAs were annotated with publicly available databases, including Gene Ontology terms (GO, http://geneontology.org/docs/go-enrichment-analysis/) and KEGG (https://www.genome.jp/kegg/pathway.html) databases. The functional enrichment results of genes with *P* < 0.05 were considered as significant.

### Construction of interaction network for heat shock proteins (HSPs) encoding DEGs and DElncRNAs

All DEGs encoding HSPs were searched in the DEGs list and annotated by the ClueGO software [92]. The interaction networks among DEGs encoding HSPs and their interacting DElncRNAs were constructed and visualized by the Cytoscape version 3.7.2 [93].

## Supplementary Information

**Additional file 1 : Table S1.** Primers used for real-time quantitative PCR (RT-qPCR) experiment of lncRNAs.

**Additional file 2 : Table S2.** Statistics summary of reads generated from 12 RNA-seq libraries in Control and H120 groups.

**Additional file 3 : Table S3.** Summary of the DElncRNAs and DEGs identified in liver and adrenal gland tissues.

**Additional file 4 Table S4.** Pearson Correlation Coefficient (PCC) analysis of DElncRNAs and DEGs identified in liver and adrenal gland tissues.

**Additional file 5 : Table S5.** GO analysis of target DEGs of DElncRNAs predicted based on *trans-*regulation. The coexpression level between DEGs and DElncRNAs met the criterion of |PCC| > 0.99 for performing the functional annotation.

**Additional file 6 : Figure S1.** The top 20 pathways from the enrichment analysis of the target DEGs in liver and adrenal glands under heat stress (H120). (A, B and C) The top 20 pathways from the enrichment analysis of the target DEGs, which were functionally predicted based on *trans*- and *cis*-regulatory action, as well as on sequence similarity method, respectively. The pathway analysis was performed by the Kyoto Encyclopedia of Genes and Genomes; Rich factor means enrichment factor.

**Additional file 7 : Table S6.** The list of target DEGs of DElncRNAs predicted by *cis*-regulatory action in liver and adrenal gland tissues.

**Additional file 8 : Table S7.** GO analysis of target genes of DElncRNAs predicted by *cis*-regulatory action in liver and adrenal gland tissues. The DElncRNA-mRNA pairs located on the same chromosome within 100 kb were assumed as potentially *cis*-regulated.

**Additional file 9 : Table S8.** GO analysis of target DEGs of DElncRNAs predicted by *cis*-regulatory action in liver and adrenal gland tissues. The DElncRNA-mRNA pairs located on the same chromosome within 100 kb were assumed as potentially *cis*-regulated.

**Additional file 10 : Table S9.** The list of target genes of DElncRNAs in liver and adrenal glands predicted based on the sequence similarity search method.

**Additional file 11 : Table S10.** GO analysis of target genes of DElncRNAs predicted based on the sequence similarity.

**Additional file 12 : Table S11.** GO analysis of target DEGs of DElncRNAs predicted based on the sequence similarity.

## Data Availability

The strand-specific RNA sequencing datasets generated during the current study are available in the Sequence Read Archive (SRA) database at the National Center for Biotechnology Information (NCBI) with the BioProject ID PRJNA624751.

## Abbreviations

HS: Heat stress
lncRNAs: Long-non-coding RNAs
H120: 42 °C for 120 min
DElncRNAs: Differentially expressed lncRNAs
DEGs: Differentially expressed genes
HSPs: Heat shock proteins
PBS: Phosphate buffer solution
FPKM: Fragments per kilobase of exon model per million reads mapped values
PCG: Protein-coding gene
NCBI NR protein database: National Center for Biotechnology Information non-redundant protein database
UniProt: Universal Protein Resource
CNCI: Coding-non-coding index
PLEK: K-mer scheme
CPAT: Coding potential assessment tool
RT-qPCR: Real-Time Quantitative PCR
GAPDH: Glyceraldehyde-3-phosphate dehydrogenase
Ct: Threshold cycle
PCC: Pearson correlation coefficient
GO: Gene Ontology terms
KEGG: Kyoto Encyclopedia of Genes and Genomes
BP: Biological process
CC: Cellular component
MF: Molecular function
hsrω: Hsr-omega
HSR1: Heat shock RNA-1
