# Supplementary figures and images for: Genome-wide identification and functional prediction of long non-coding RNAs in Sprague-Dawley rats during heat stress
Source: BMC Genomics. 2021 Feb 17;22:122. doi: 10.1186/s12864-021-07421-8 (PMC7891137; doi:10.1186/s12864-021-07421-8)

A

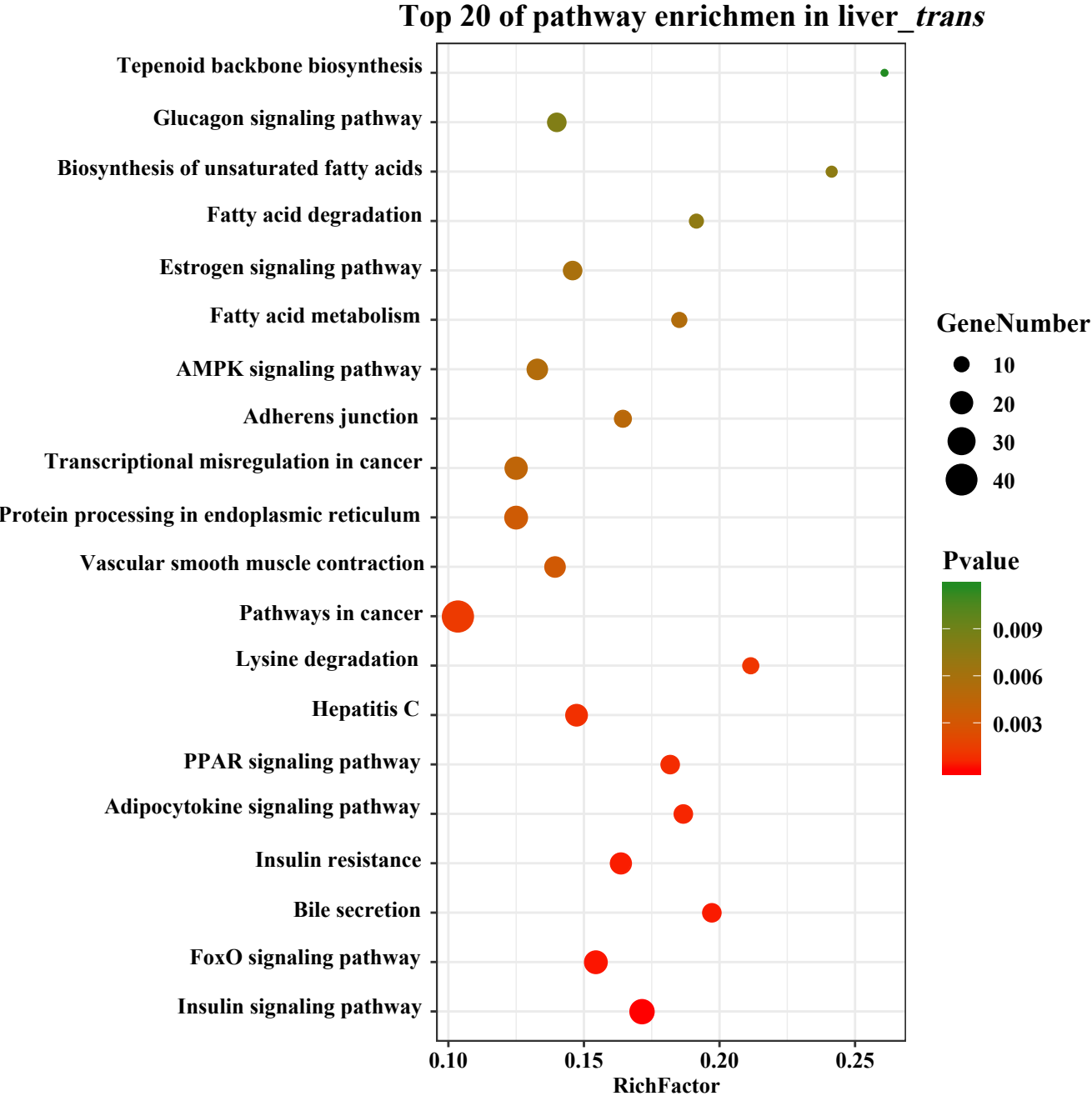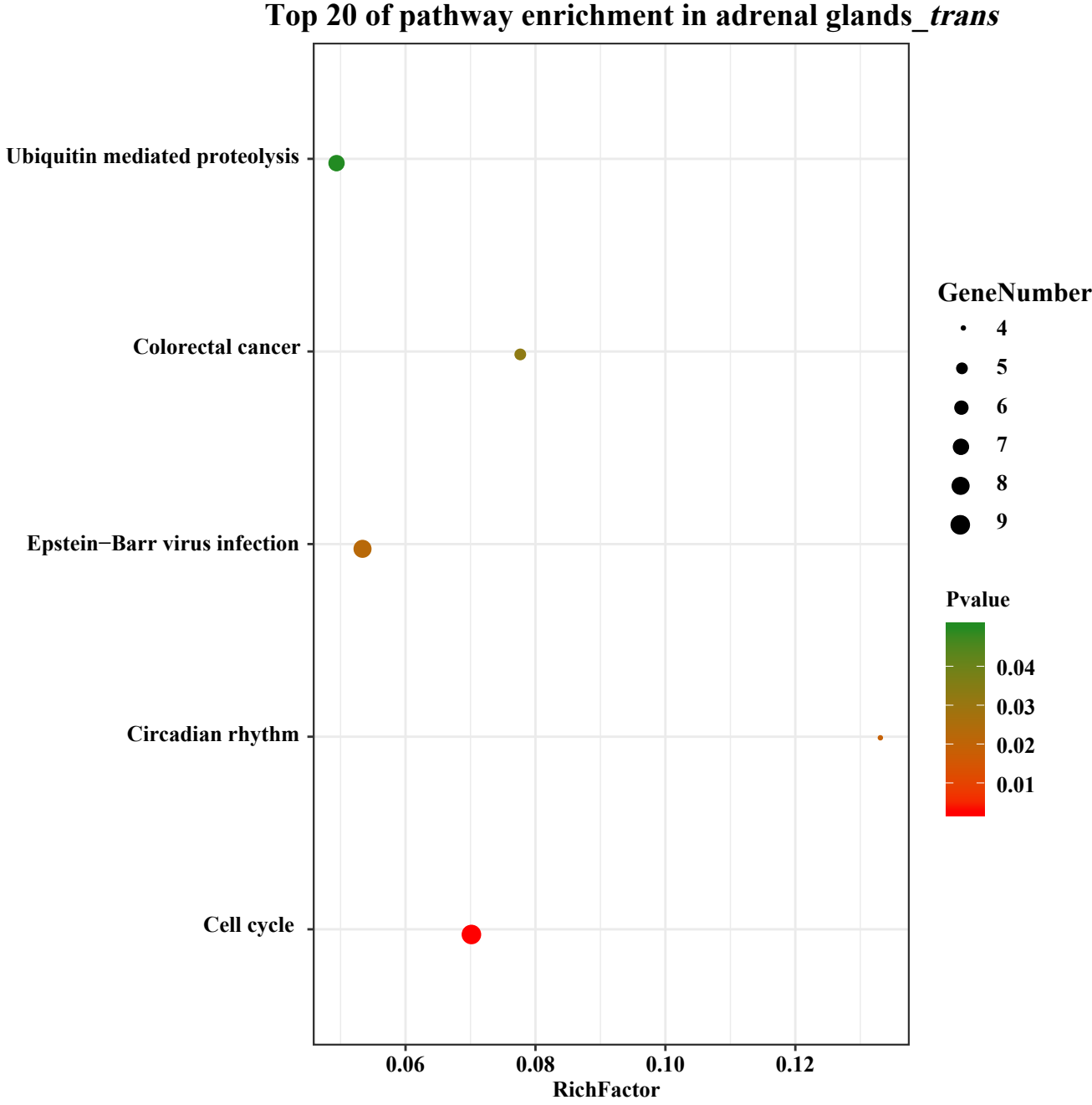

B

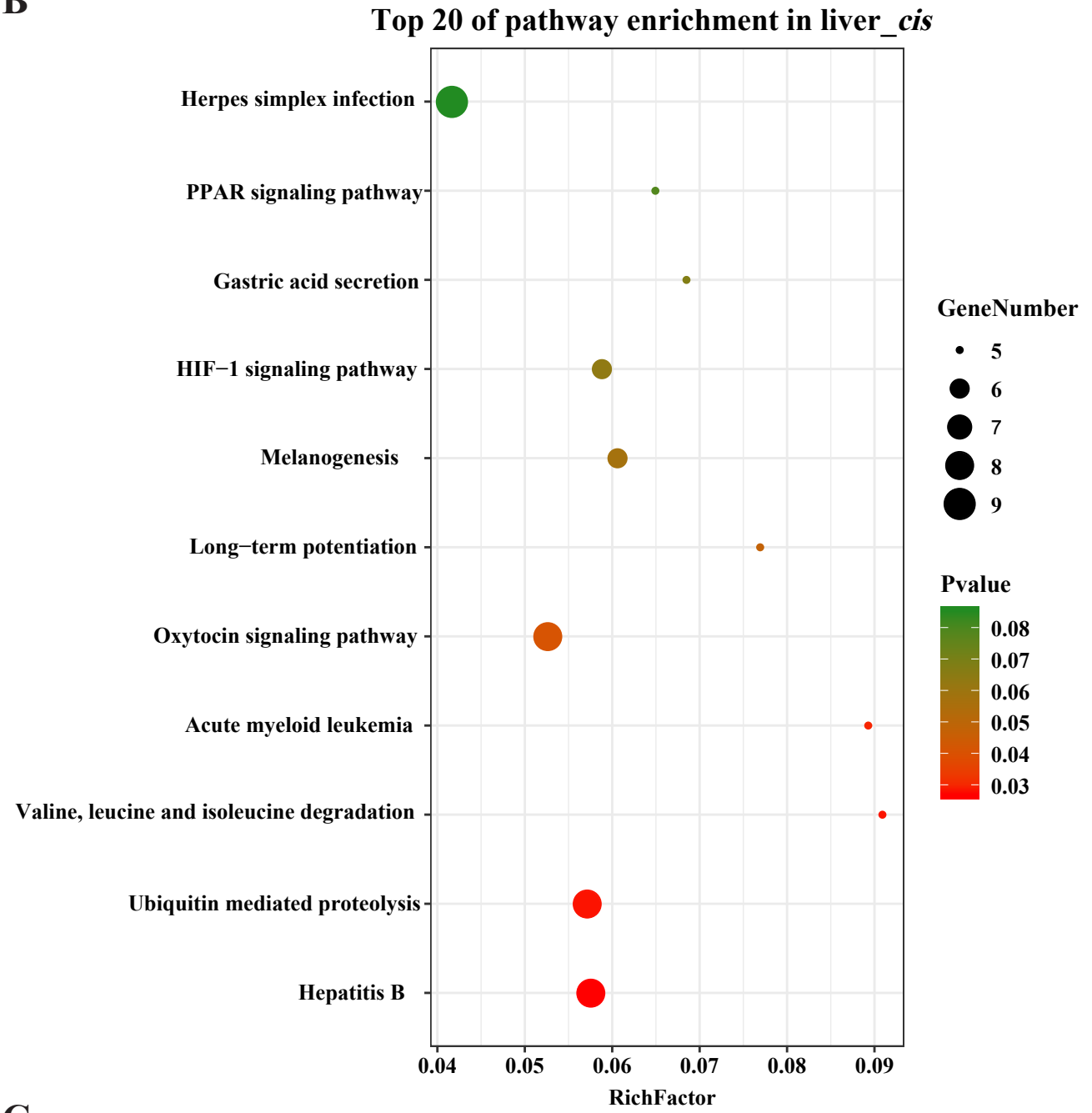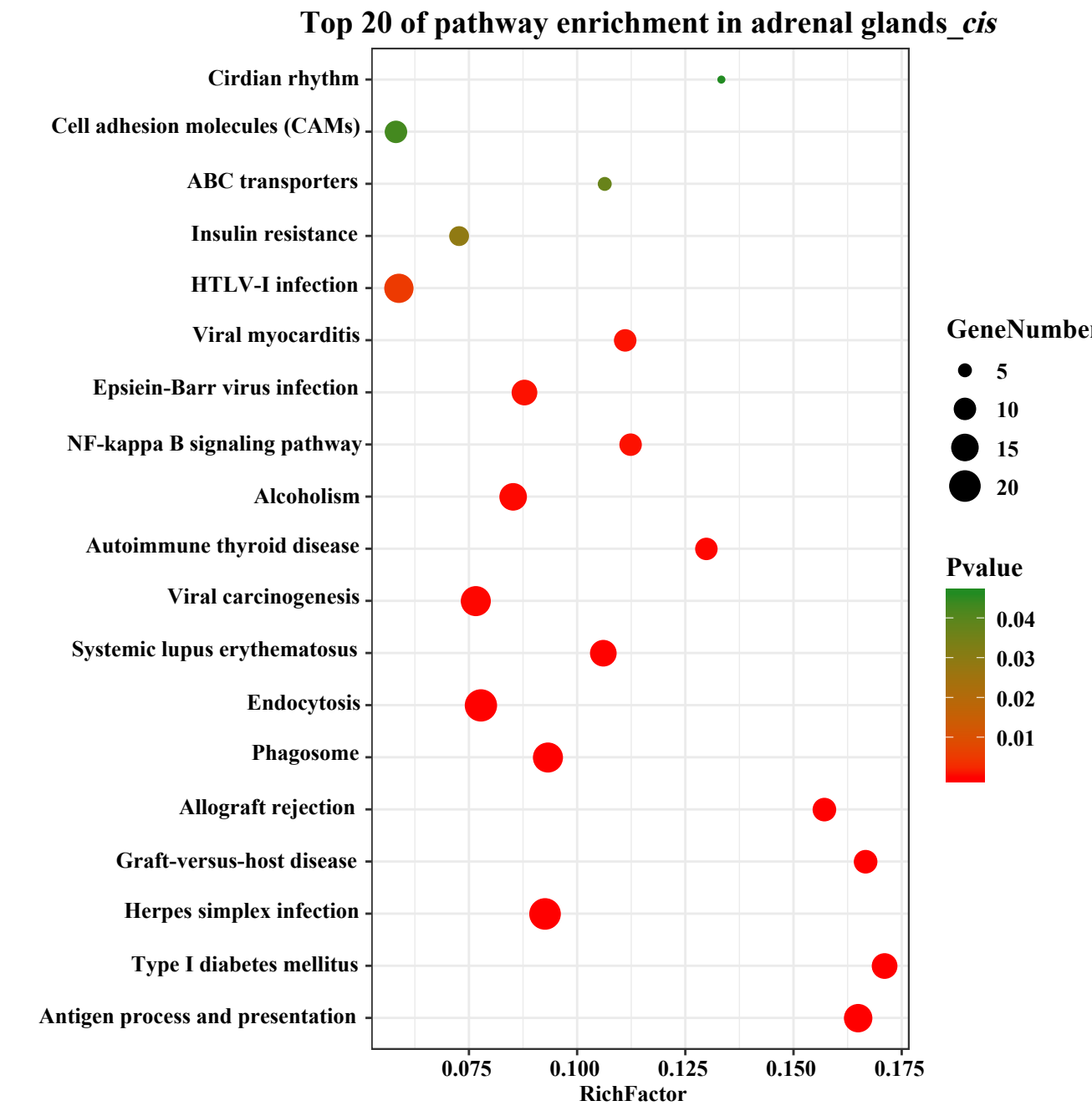

C

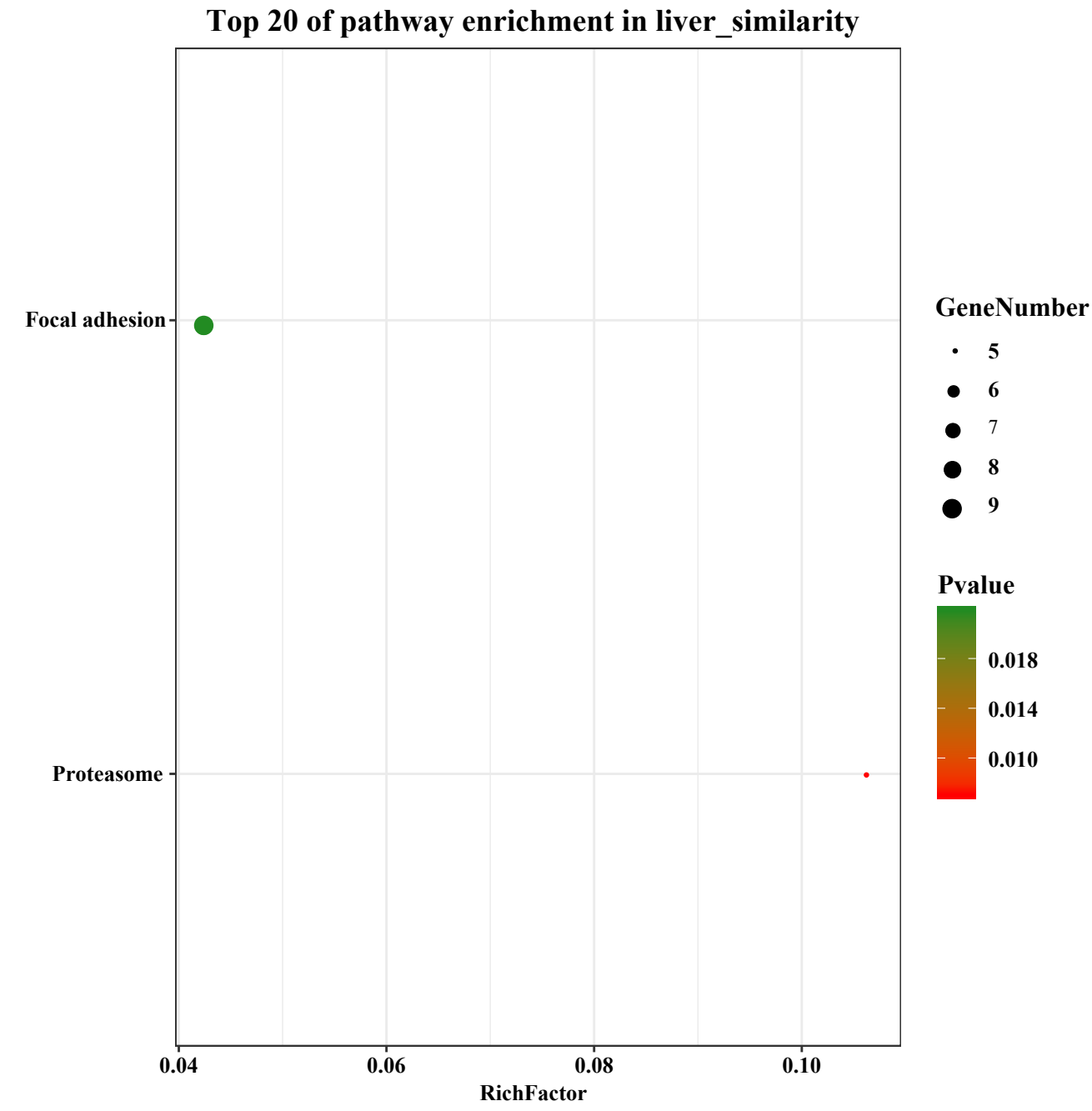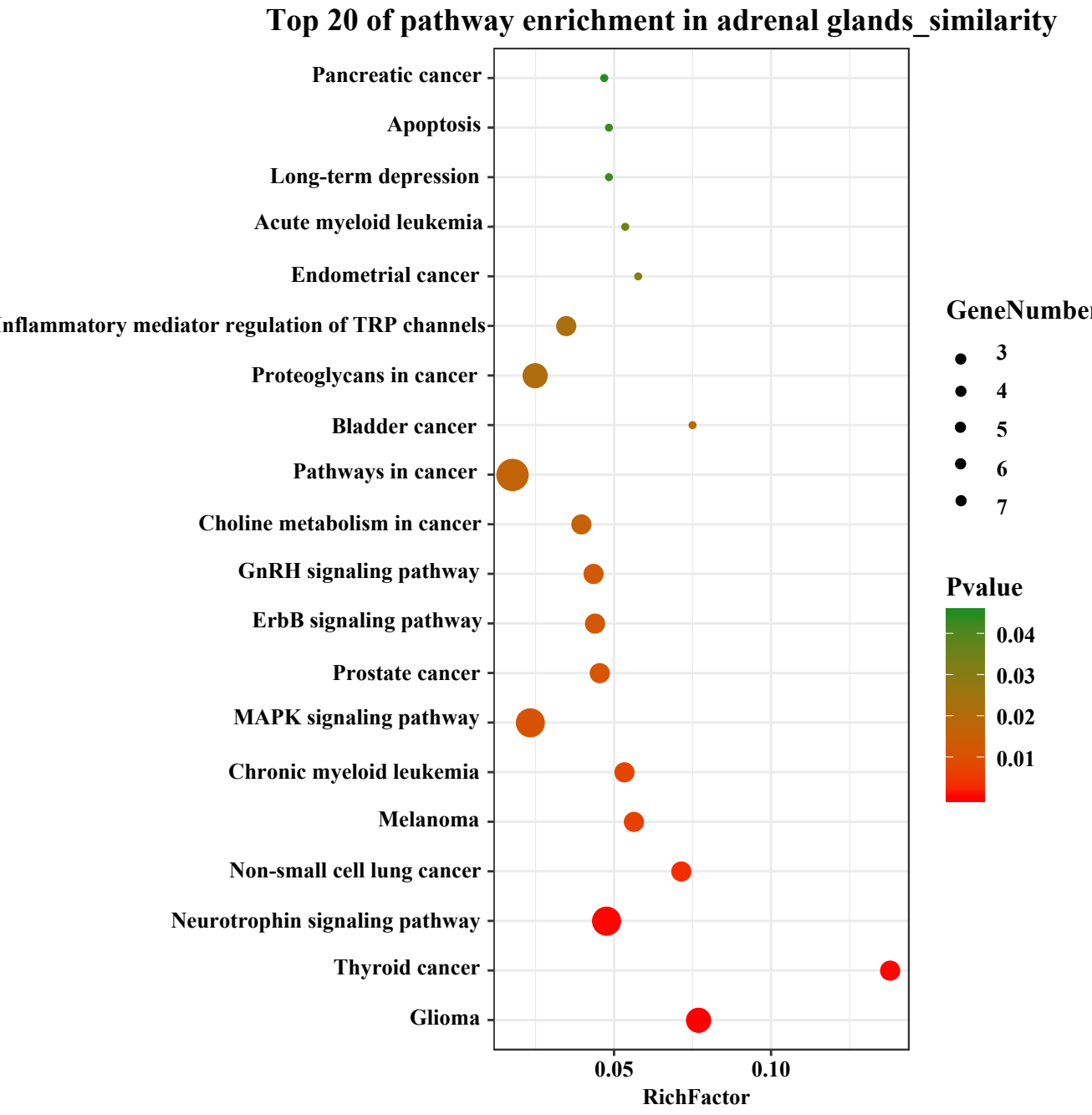

Supplement: Supplementary file 6 — Additional file 6 : Figure S1. The top 20 pathways from the enrichment analysis of the target DEGs in liver and adrenal glands under heat stress (H120). (A, B and C) The top 20 pathways from the enrichment analysis of the target DEGs, which were functionally predicted based on trans- and cis-regulatory action, as well as on sequence similarity method, respectively. The pathway analysis was performed by the Kyoto Encyclopedia of Genes and Genomes; Rich factor means enrichment factor. [file 12864_2021_7421_MOESM6_ESM.pdf]
